# Editorial: International Day of Happiness 2022: public mental health

**DOI:** 10.3389/fpubh.2024.1413970

**Published:** 2024-05-02

**Authors:** Andrew T. Olagunju

**Affiliations:** ^1^Forensic Psychiatry Program, St. Joseph's Healthcare, Hamilton, ON, Canada; ^2^Department of Psychiatry and Behavioural Neurosciences, McMaster University, Hamilton, ON, Canada; ^3^Discipline of Psychiatry, The University of Adelaide, North Terrace, Adelaide, SA, Australia

**Keywords:** global health, happiness, mental health, quality of life, wellbeing

In 2012, the United Nations (UN) passed a resolution to proclaim March 20th of every year as the International Day of Happiness, partly in recognition of the significance of happiness to humans and the UN economic agenda (1). Through this action, the UN highlights the importance of happiness to wellbeing, creates better awareness about the value of the “happiness-wellbeing paradigm” to healthy living and encourages everyone to continue to pursue it.

Although the pursuit of happiness has been a prime subject of interest to humans from time immemorial, it was not until recently that scientific research started to focus on exploring the multifaceted dimensions of happiness to better understand its contributions or relationship with wellbeing, quality of life and meaningful living (2). There is evidence to suggest that academic and research work on happiness is gaining traction, especially in the field of positive psychology and psychiatry to better understand its intersection with emotional wellbeing, quality of life and positive psychosocial characteristics (3). In support of the benefits of equitable mental health services on happiness and wellbeing, this Research Topic consists of a collection of articles, highlighting interesting public mental health issues and provisions among different global populations to promote happy living and health for all. Specifically, the articles included in the Research Topic addressed diverse issues ranging from mental health burdens in diversified settings or populations and gaps in mental health services to interventions with potential benefits on wellbeing, and quality of life.

Amini et al. addressed the extended impacts of caregiving for individuals with chronic mental illnesses on family caregivers in Iran. While all groups of caregivers recruited into the study expressed a perceived need for various forms of support, parents or children with families with chronic mental illness indicated a greater need for support. The most affected areas of need identified by family caregivers pertain to the need for better information about the illness, especially on how to care for their relatives, access social resources, use the available support and mitigate expressed emotion. Zhong et al. highlighted the burden and gap in mental health services among people living with vision disability in China. In their work among displaced individuals in Vanuatu, Nzayisenga et al. highlight the benefits of traditional, group and professional support to addressing public mental health needs among vulnerable and displaced people post-disaster. Conversely, the other papers explored the impacts of lifestyle and physical activities on mental wellbeing with some interesting findings identified. For example, in a cohort of college students in China, Zhang et al. showed that lifestyle behaviors and coping styles are both predictors of mental health. Similarly, Li et al. reported that frequent physical activity was linked with improvement in happiness via enhancement of mental and overall health conditions among people interviewed in the Chinese General Social Survey. Lastly, Keyes et al. noted that attendance of life sporting events was positively associated with improvement in subjective wellbeing and was protective against loneliness in a large sample study of individuals in the United Kingdom.

Overall, the articles published in this Research Topic highlight important perspectives on multiple aspects of public mental health to revisit the major contributions of mental wellbeing to health and wellbeing. Addressing the gaps in mental health delivery with an equitable lens and the promotion of innovative strategies are critical for attaining global health and the pursuit of wellbeing and happiness for all.

## Author contributions

AO: Conceptualization, Formal analysis, Investigation, Methodology, Resources, Validation, Visualization, Writing – original draft.
